# Advanced Risk Stratification in Non-Ischemic Cardiomyopathy: The Prognostic Role of Cardiac Magnetic Resonance

**DOI:** 10.3390/jcm15020841

**Published:** 2026-01-20

**Authors:** Guido Pastorini, Marzia Testa, Eleonora Indolfi, Enrica Conte, Fabio Anastasio, Mauro Feola

**Affiliations:** 1 Cardiology Division, Ospedale Regina Montis Regalis Strada del Rocchetto 99, ASLCN1, 12084 Mondovi, Italy; 2 Cardiology Division, Ospedale Dario Camberlingo, ASL, 72017 Brindisi, Italy; 3 Cardiovascular Rehabilitation, Ospedale Fossano ASLCN1, 12045 Fossano, Italy

**Keywords:** cardiac magnetic resonance, mapping, non-ischemic cardiomyopathy

## Abstract

Cardiac magnetic resonance (CMR) imaging has been considered crucial in non-ischemic cardiomyopathy (NICM). This study aims to evaluate the role of CMR in identifying risk factors for life-threatening events in patients with NICM and reduced left ventricular ejection fraction (LVEF). **Methods:** We analysed 57 (mean age 62.5 ± 11.4 years, 68.4% male) first-diagnosed NICM patients with reduced LVEF (mean 42 ± 9%). CMR assessments evaluated LVEF, right ventricular ejection fraction (RVEF), cardiac T1 mapping, extracellular volume (ECV), and the presence/extension of late gadolinium enhancement (LGE). Patients were monitored for a composite endpoint including sudden cardiac death (SCD), major ventricular arrhythmic events, and hospitalization for heart failure (HHF). **Results:** During a median follow-up lasting 543 days, 18 patients (31%) experienced cardiovascular events. A higher native T1 mapping value (1076 (1025–1120) ms vs. 999 (990–1037) ms, *p* < 0.001), a higher ECV (34 ± 6% vs. 28 ± 4,% *p* < 0.001) and a reduced RVEF (52 ± 13% vs. 60 ± 9%, *p* < 0.03) proved to be significantly correlated to an increased HHF, arrhythmic and SCD risk. Additionally, a native T1 mapping value exceeding 1018 ms demonstrated an increased risk (HR: 6.285; 95% CI: 2.044–19.326, *p* = 0.001) as well as an ECV greater than 28% (HR: 19.752; 95% CI: 2.622–148.817, *p* = 0.004) for composite endpoint. **Conclusion:** In NICM patients, elevated native T1 mapping and ECV values identified a high-risk subgroup for arrhythmic events while LVEF, and RVEF provide further risk stratification for the composite endpoint. CMR assessment may optimize risk stratification in NICM patients.

## 1. Introduction

NICM represents a significant global health concern, recognized as a leading cause of cardiovascular morbidity and mortality [1,2]. This condition is characterized by left ventricular (LV) dilation and reduced LV systolic function, associated with a poor prognosis: reported 1-year mortality rates of 25–30% and 5-year rates reaching up to 50% [3]. Patients with NICM are at a notable risk for early mortality primarily due to ventricular arrhythmias (VA) and progressive heart failure [2,4,5,6]. Historically, physicians have relied heavily on LVEF and functional capacity to assess the risk of SCD in heart failure patients, including those with NICM [5,7]. Current guidelines typically recommend implantable cardioverter-defibrillator (ICD) therapy for primary prevention in patients with symptomatic heart failure and an LVEF ≤ 35% [4,5,6]. However, LVEF alone lacks sufficient sensitivity and specificity for precise prediction of ventricular arrhythmic events, having no incremental prognostic value for SCD beyond other clinical parameters [4,5]. This highlights an urgent need to refine risk stratification algorithms to more accurately identify patients at the highest risk of life-threatening events [4].

In recent years, CMR imaging has gained recognition as a pivotal tool for the comprehensive evaluation of patients with heart failure and cardiomyopathies [6,8,9]. CMR provides detailed insights into myocardial structure and function and it serves as the gold standard for quantifying chamber size and ejection fraction [8,9]. Beyond conventional assessment, advanced tissue characterization techniques such as LGE, T1 mapping, and ECV fraction measurement offer crucial diagnostic and prognostic information by visualizing and quantifying myocardial tissue composition [9]. This single-centre study aimed to evaluate the role of CMR in identifying risk factors for life-threatening events in patients with NICM and reduced LVEF.

## 2. Materials and Methods

This is a retrospective observational study including consecutive patients with newly diagnosed NICM referred to our Hospital between January 2020 and December 2022. NICM was defined as LV systolic dysfunction (LVEF < 50%) in the absence of obstructive coronary artery disease (≥50% stenosis) or prior myocardial infarction as confirmed by invasive coronary angiography or coronary computed tomography angiography. Exclusion criteria were contraindications to cardiac magnetic resonance (CMR), severe renal impairment (estimated glomerular filtration rate < 30 mL/min/1.73 m^2^), or pre-existing implantable cardioverter-defibrillator (ICD). The study protocol was approved by the institutional ethics committee (Ethical Committee n.10–18 in 2018) and all participants provided written informed consent.

CMR imaging was performed using a 1.5 T scanner (Philips Ingenia) with a standardized protocol. Cine steady-state free precession (SSFP) sequences acquired bi-ventricular volumes and function in standard views. Native T1 mapping was performed using a motion-corrected modified Look-Locker inversion recovery (MOLLI) sequence (5(3)3 scheme) in mid-ventricular short-axis slices prior to contrast administration. Post-contrast T1 mapping was repeated 15–20 min after gadobutrol (0.15 mmol/kg) injection using an identical acquisition scheme. The ECV fraction was calculated using the formula: (ΔR1 myocardium/ΔR1 blood) × (1 − hematocrit), where R1 = 1/T1. To ensure accurate calculation, hematocrit was measured in all patients within 7 days of the CMR examination. LGE imaging was acquired 5 min post-contrast using phase-sensitive inversion-recovery SSFP sequences. LGE extent was quantified as the percentage of myocardial mass using semi-automated thresholding (5 SD above remote myocardium).

Image analysis was conducted offline by two experienced observers blinded to clinical outcomes using the Philips IntelliSpace Portal workstation. LVEF, RVEF, end-diastolic/systolic volumes, and LV mass were derived from manual contouring of cine stacks. T1 and ECV maps were analyzed by placing regions of interest (ROIs) in the mid-myocardium, carefully avoiding the blood pool and artifacts. 

Patients were followed until December 2024 for a composite endpoint comprising SCD, life-threatening ventricular arrhythmias (sustained ventricular tachycardia or ventricular fibrillation), and HHF. Clinical events were adjudicated by an independent committee blinded to CMR findings. A secondary arrhythmic endpoint was defined by excluding heart failure hospitalizations from the composite. Statistical analysis involved univariate Cox regression to identify predictors of adverse outcomes, with hazard ratios (HRs) and 95% confidence intervals (CIs) calculated for both continuous and categorical variables. Receiver operating characteristic (ROC) curves determined optimal cut-offs for T1 and ECV, while Kaplan–Meier survival analysis compared event-free survival across strata. Multivariable analysis was attempted but limited by the event rate; therefore, results are primarily reported as univariate associations. All statistical tests were two-sided, with significance set at *p* < 0.05 (SPSS v28.0, IBM, Chicago, IL, USA). AI assistance (Claude Sonnet 4, Anthropic) was utilized for stylistic refinement only, with no impact on study design, data analysis, or interpretation.

## 3. Results

### 3.1. Baseline Characteristics

A total of 57 patients (mean age: 62.5 ± 11.4 years, 68.4% male) with NICM and reduced LVEF (mean value: 42 ± 9%) were included in this retrospective analysis. Clinical characteristics and medical therapies of the population are described in Table 1.

During a median follow-up of 543 days [IQR: 314–791], 18 patients (31.6%) experienced the composite endpoint consisting of SCD (3/18), major ventricular arrhythmic events (5/18), or hospitalization for heart failure (12/18).

### 3.2. Comparison of Event and Non-Event Groups

Univariate analyses revealed that native T1 mapping values were significantly higher in patients who experienced adverse cardiovascular events included in the composite endpoint in comparison to those who remained event-free (1058 ± 45 ms vs. 1000 ± 34 ms, *p* < 0.001). Similarly, ECV was significantly elevated in patients with events (33 ± 6% vs. 26 ± 3%, *p* < 0.001). RVEF was also significantly lower in patients with events (52 ± 13% vs. 60 ± 9%, *p* = 0.04) Table 2.

**Figure 1:**
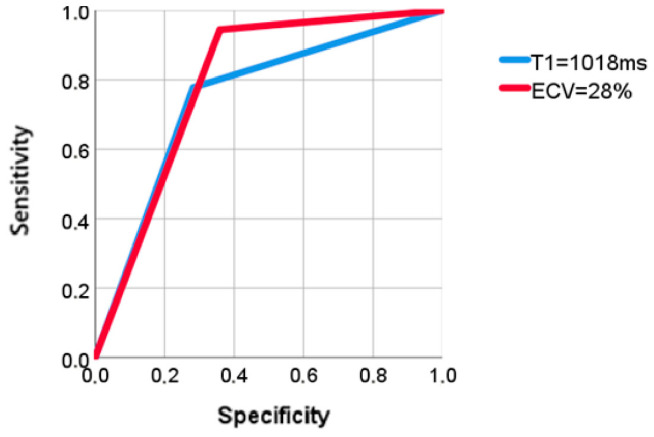
ROC Curves for Native T1 Mapping and ECV. ROC curve analysis demonstrates the discriminatory capacity of native T1 mapping (blue line, T1 = 1018 ms) and extracellular volume fraction (red line, ECV = 28%) for the prediction of adverse events in patients with non-ischemic cardiomyopathy.

ROC curve analysis (Figure 1) demonstrated that native T1 had a significant predictive value for the composite endpoint, with an area under the curve (AUC) of 0.748 (*p* = 0.001). A cut-off value of 1018 ms yielded a sensitivity of 77.8% and a specificity of 81.8%. Moreover, patients with native T1 > 1018 ms exhibited a significantly increased risk of the composite endpoint (HR: 6.285; 95% CI: 2.044–19.326, *p* = 0.001) and SCD/arrhythmic events alone (HR: 5.390; 95% CI: 1.083–26.822, *p* = 0.040). Consistent with these findings, Kaplan–Meier survival analysis confirmed a statistically significant difference in event-free survival stratified by the T1 threshold of 1018 ms (log-rank test: χ^2^ = 13.344, *p* < 0.001). Furthermore, ECV demonstrated robust prognostic value. ROC analysis yielded an AUC of 0.793 (*p* < 0.001) for ECV in predicting the composite endpoint. An ECV threshold of 28% showed high specificity (94.9%) and high sensitivity (94.4%) for the composite outcome. Patients with ECV > 28% exhibited a markedly increased risk of adverse events (HR: 19.752; 95% CI: 2.622–148.817, *p* = 0.004). Notably, a value of ECV > 34% showed a higher HR for SCD and arrhythmic events (HR: 77.795; 95% CI: 0.254–23,791.956, *p* = 0.136), although this did not reach statistical significance. The survival analysis further confirmed a significant difference in event-free survival between groups stratified by ECV (log-rank test: χ^2^ = 16.621, *p* < 0.001). Additionally, lower RVEF was associated with the composite endpoint (RVEF: 52 ± 13 vs. 60 ± 9, *p* = 0.04). However, other RV parameters—such as RV end-diastolic volume (RVEDV), RV stroke volume (RVSV), and presence of RV hypertrophy or other structural abnormalities—did not show significant differences between event and non-event groups. In our cohort, although LV parameters including LVEDV index, LVSV index, LV mass, LGE extent and presence were significantly higher in patients who experienced the composite endpoint, they did not show a statistically significant correlation with isolated arrhythmic events or SCD (LVEDV index: *p* = 0.36; LVSV index: *p* = 0.27; LV mass: *p* = 0.61; LGE mass: *p*= 0.66; LGE presence: *p* = 0.64).

## 4. Discussion

This retrospective analysis underscores the prognostic power of CMR imaging (specifically T1 mapping, ECV and functional parameters) in the risk stratification of life-threatening arrhythmic events and heart failure hospitalization in NICM patients. LGE imaging is a well-established method for detecting focal myocardial fibrosis and replacement scar, which are pathological hallmarks of NICM that can predispose to malignant ventricular arrhythmia and SCD [1,3]. The presence and extent of myocardial scar detected by LGE have consistently shown strong and independent associations with adverse cardiovascular outcomes, including all-cause mortality, cardiac death, and particularly SCD and appropriate ICD shocks in NICM patients [2,5]. Specific LGE patterns, such as patchy mid-wall striae or a ring-like distribution, have also been correlated with an increased risk of arrhythmic events [7,10]. Despite its utility, a significant limitation of conventional LGE is its inability to characterize diffuse myocardial fibrosis, which may affect the myocardium uniformly and go undetected in the absence of “normal” myocardium for comparison [3]. To address this limitation, myocardial T1 mapping and the derived ECV fraction have emerged as essential quantitative CMR techniques [3,6]. These methods allow for the non-invasive detection and quantification of diffuse myocardial abnormalities and interstitial fibrosis, which are critical pathophysiological processes in NICM [9,11]. In line with previous literature, we found that native T1 values are prolonged and ECV values are significantly increased in NICM patients compared to healthy controls [2,11]. These findings highlight the incremental value of native T1 mapping and ECV as robust imaging biomarkers for risk stratification in NICM. Elevated values of both parameters identify a high-risk subgroup for life-threatening arrhythmic events and SCD, suggesting their potential role in guiding clinical decision-making. While LGE remains valuable for detecting focal fibrosis, it relies on the subjective identification of a relative difference in gadolinium uptake between the enhancing fibrotic areas and adjacent “normal” nulled myocardium. Consequently, it may be less sensitive for capturing the diffuse interstitial fibrosis often found in NICM. In contrast, T1 mapping and ECV provide a quantitative and more sensitive assessment of diffuse myocardial fibrosis without requiring a reference ‘normal’ tissue. Diffuse fibrosis appears to be a critical factor in risk stratification for adverse cardiac outcomes, particularly in NICM offering greater reproducibility and fewer operator-dependent variations. Importantly, both native T1 and ECV have demonstrated significant prognostic value for major adverse cardiac events (MACE), encompassing all-cause mortality, heart failure-related hospitalizations, and ventricular arrhythmias [12]. Specifically, an ECV ≥ 30% has been identified as a strong independent predictor of adverse arrhythmic events, and it has shown the ability to discriminate arrhythmic risk even among patients with LGE presence or an LVEF ≤ 35% [6]. Native T1 may also play a crucial role, potentially being more useful for selecting patients eligible for ICDs as it has been independently associated with arrhythmia-related events. This clinical study aims to further evaluate the comprehensive role of CMR imaging, including LGE, T1 mapping, and ECV quantification, in identifying specific risk factors for life-threatening events in patients with NICM, particularly those with a reduced LVEF. By leveraging these advanced imaging biomarkers, we seek to improve current risk stratification strategies and ultimately optimize clinical management and patient outcomes in this vulnerable population. By integrating quantitative myocardial fibrosis markers, native T1 mapping and ECV), we identified distinct and clinically translatable thresholds (T1 > 1018 ms, ECV > 28%) that robustly predict adverse outcomes, offering actionable criteria for clinical decision-making. These findings, built upon prior evidence [13,14], introduce critical advancements that distinguish our work from existing literature. A key strength of this study lies in its focus on a rigorously phenotype NICM cohort, excluding ischemic etiologies through coronary angiography or CT, thereby isolating the pure prognostic value of CMR biomarkers in this population—addressing a limitation in prior studies that included mixed cardiomyopathy groups [15]. Unlike previous investigations emphasizing continuous T1/ECV values, we define precise, clinically translatable thresholds with high specificity and sensitivity, enabling immediate application in risk stratification protocols. This aligns with emerging evidence underscoring the RV’s role in cardiomyopathy progression [13].

## 5. Conclusions

Our findings advocate for the routine incorporation of parametric mapping and RV assessment in CMR protocols for NICM patients. A complete CMR examination might help in prediction of adverse cardiovascular events (arrhythmic or heart failure hospitalization). Future prospective studies will create and validate risk scores based on T1 mapping and ECV parameters exploring the mechanistic interplay between diffuse fibrosis, RV dysfunction, and arrhythmogenesis. This observational study presents some limitations. Its retrospective design and single-centre enrolment inherently limit widespread utilization, while the relatively small sample size may underpower analyses for rare outcomes such as isolated SCD: due to the small sample size and exploratory nature of this study, these findings should be considered hypothesis-generating and require further validation before being implemented in clinical decision-making. The T1 and ECV thresholds, although highly specific, require validation in larger, multicenter cohorts to confirm its reproducibility across scanners and populations. Additionally, the lack of association between LGE extent and arrhythmic risk in our cohort warrants caution, as prior studies have reported conflicting results [14]. Furthermore, our study focused primarily on CMR-derived parameters; future research could investigate whether combining tissue characterization with innovative anthropometric indices, such as those reflecting thoracic morphology (modified Haller index), could further refine risk stratification in this population. These findings should be considered hypothesis-generating and require further validation before being implemented in clinical decision-making. Finally, the absence of histopathological correlation for fibrosis remains a limitation, though this is a common constraint in clinical CMR research.

## Figures and Tables

**Table 1:** Baseline Characteristics of the Study Population Stratified by Event Status.

Variable	Total (57)	Event (18)	No Event (39)	*p*-Value
Age (years)	62.5 ± 11.4	59.7 ± 9.2	63.7 ± 12.2	0.038
Height (cm)	173.00 [167.00–180.00]	175.00 [168.50–179.50]	172.00 [165.00–180.00]	0.441
Weight (kg)	74.00 [61.00–83.00]	76.00 [62.00–83.50]	74.00 [62.00–82.00]	0.811
Body Surface Area	1.90 [1.72–2.00]	1.93 [1.79–1.97]	1.90 [1.72–2.00]	0.597
Body Mass Index	24.24 [21.62–27.38]	23.76 [21.40–28.38]	24.69 [21.82–26.88]	0.521
Male Sex	39 (68.4%)	12 (66.7%)	27 (69.2%)	1.000
Left Bundle Branch Block	9 (15.8%)	2 (11.1%)	7 (17.9%)	0.697
Hypertension	36 (63.2%)	13 (72.2%)	23 (59.0%)	0.354
Hypercholesterolemia	40 (70.2%)	11 (61.1%)	29 (74.4%)	0.316
Familial History of CAD	4 (7.0%)	1 (5.6%)	3 (7.7%)	1.000
Diabetes	7 (12.3%)	0 (0.0%)	7 (17.9%)	0.138
Smoker	4 (7.0%)	2 (11.1%)	2 (5.1%)	0.589
Familial History of SCD	3 (5.3%)	0 (0.0%)	3 (7.7%)	0.551
Chest Pain	2 (3.5%)	0 (0.0%)	2 (5.1%)	1.000
Dyspnea	32 (56.1%)	12 (66.7%)	20 (51.3%)	0.315
Beta-blocker	33 (57.9%)	10 (55.6%)	23 (59.0%)	1.000
ACE-inhibitor	26 (45.6%)	10 (55.6%)	16 (41.0%)	0.380
Angiotensin Receptor Blockers	9 (15.8%)	3 (16.7%)	6 (15.4%)	1.000
Angiotensin Receptor-Neprilysin Inhibitor	3 (5.3%)	2 (11.1%)	1 (2.6%)	0.222
SGLT2 Inhibitor	2 (3.5%)	0 (0.0%)	2 (5.1%)	1.000
Mineralocorticoid Receptor Antagonist	7 (12.3%)	3 (16.7%)	4 (10.3%)	0.686
Atrial Fibrillation	11 (19.3%)	6 (33.3%)	5 (12.8%)	0.117

Data are presented as mean ± standard deviation for normally distributed continuous variables, median [interquartile range] for non-normally distributed continuous variables, and count (n) with percentage (%) for categorical variables. *p*-values compare the Event = 1 group vs. the Event = 0 group. *p* < 0.05 indicates statistical significance. Abbreviations: CAD, Coronary Artery Disease; SCD, Sudden Cardiac Death.

**Table 2:** Baseline clinical and CMR characteristics of the study population, stratified by composite endpoint occurrence.

	Total (57)	Event (18)	No Event (39)	*p*-Value
Age (years)	62.5 ± 11.4	59.7 ± 9.2	63.7 ± 12.2	0.17
Male sex, n (%)	39 (68.4%)	12 (66.7%)	27 (69.2%)	0.53
BMI (kg/m^2^)	24.3 ± 3.8	24.2 ± 4.1	24.3 ± 3.8	0.97
Follow-up (days)	543 [314–791]	119 [69–317]	690 [494–935]	<0.001
LVEDV index (mL/m^2^)	104 [83–118]	124 [94–150]	97 [82–113]	0.007
Abnormal LVEDV index, n (%)	33 (57.9%)	13 (72.2%)	20 (51.3%)	0.16
LVSV index (mL/m^2^)	79 [65–90]	76 ± 24	81 ± 20	0.40
Abnormal LVSV index, n (%)	12 (21.1%)	7 (38.9%)	5 (12.8%)	0.03
LVEF (%)	42 ± 9	35 ± 8	46 ± 7	<0.001
Abnormal LVEF (%)	44 (77.2%)	18 (100%)	26 (66.7%)	0.005
LVMASS (g)	123 ± 37	139 ± 41	116 ± 33	0.05
Abnormal LVMASS, n (%)	8 (14.0%)	6 (33.3%)	2 (5.1%)	0.009
RVEDV (ml)	77 ± 20	81 ± 24	74 ± 17	0.26
Abnormal RVEDV index, n (%)	7 (12.3%)	3 (16.7%)	4 (10.3%)	0.66
RVSV (mL)	79 ± 20	77 ± 22	60 ± 9	0.54
Abnormal RVSV index, n (%)	5 (8.9%)	3 (16.7%)	2 (5.1%)	0.31
RVEF (%)	57 ± 10	52 ± 13	60 ± 9	0.04
Abnormal RVEF, n (%)	17 (29.8%)	7 (38.9%)	10 (25.6%)	0.24
RV hypertrophy, n (%)	2 (3.5%)	1 (5.6%)	1 (2.6%)	0.53
RV abnormality, n (%)	16 (28.1%)	8 (44.4%)	8 (20.5%)	0.11
LGE mass (g)	4.0 [0.5–9.1]	9.1 [3.7–21.0]	3.0 [0.2–5.5]	0.001
LGE presence, n (%)	46 (80.7%)	16 (88.9%)	30 (76.9%)	0.47
LGE mass %	3.1 [0.6–6.1]	5.9 [2.2–14.4]	2.2 [0.3–4.7]	0.004
LGE 15%, n (%)	5 (8.9%)	4 (22.2%)	1 (2.6%)	0.03
Native T1 (ms)	1018 ± 46	1058 ± 45	1000 ± 34	<0.001
Junctional (RV insertion point) LGE, n (%)	23 (41.1%)	7 (38.9%)	16 (41%)	0.52
Edema, n (%)	10 (17.5%)	5 (27.8%)	5 (12.8%)	0.26
Non-compaction, n (%)	3 (5.3%)	0	3 (7.7%)	0.54
ECV (%)	28 ± 5	33 ± 6	26 ± 3	<0.001

Data are presented as mean ± standard deviation for normally distributed continuous variables, median [interquartile range] for non-normally distributed continuous variables, and count (n) with percentage (%) for categorical variables. “LGE mass (g)” refers to the absolute quantification of fibrosis in grams, whereas “LGE mass (%)” represents the fibrotic burden relative to the total left ventricular mass. Abbreviations: BMI, body mass index; CMR, cardiac magnetic resonance; ECV, extracellular volume; LGE, late gadolinium enhancement; LVEDV, left ventricular end-diastolic volume; LVEF, left ventricular ejection fraction; LVMASS, left ventricular mass; LVSV, left ventricular stroke volume; RVEDV, right ventricular end-diastolic volume; RVEF, right ventricular ejection fraction; RVSV, right ventricular stroke volume.

## Data Availability

The data that supports the findings of this study are available from the corresponding author upon reasonable request, subject to privacy and ethical restrictions.
